# An Italian individual-level data study investigating on the association between air pollution exposure and Covid-19 severity in primary-care setting

**DOI:** 10.1186/s12889-021-10949-9

**Published:** 2021-05-12

**Authors:** Valeria Pegoraro, Franca Heiman, Antonella Levante, Duccio Urbinati, Ilaria Peduto

**Affiliations:** IQVIA Solutions Italy S.r.l., RWS, Via Fabio Filzi 29, 20124 Milan, Italy

**Keywords:** Covid-19, Pneumonia, Air pollution, Particulate matter, Individual-level data, Primary-care

## Abstract

**Background:**

Several studies have been focusing on the potential role of atmospheric pollutants in the diffusion and impact on health of Covid-19. This study’s objective was to estimate the association between ≤10 μm diameter particulate matter (PM_10_) exposure and the likelihood of experiencing pneumonia due to Covid-19 using individual-level data in Italy.

**Methods:**

Information on Covid-19 patients was retrieved from the Italian IQVIA® Longitudinal Patient Database (LPD), a computerized network of general practitioners (GPs) including anonymous data on patients’ consultations and treatments. All patients with a Covid-19 diagnosis during March 18th, 2020 – June 30th, 2020 were included in the study. The date of first Covid-19 registration was the starting point of the 3-month follow-up (Index Date). Patients were classified based on Covid-19-related pneumonia registrations on the Index date and/or during follow-up presence/absence. Each patient was assigned individual exposure by calculating average PM_10_ during the 30-day period preceding the Index Date, and according to GP’s office province. A multiple generalized linear mixed model, mixed-effects logistic regression, was used to assess the association between PM_10_ exposure tertiles and the likelihood of experiencing pneumonia.

**Results:**

Among 6483 Covid-19 patients included, 1079 (16.6%) had a diagnosis of pneumonia. Pneumonia patients were older, more frequently men, more health-impaired, and had a higher individual-level exposure to PM_10_ during the month preceding Covid-19 diagnosis. The mixed-effects model showed that patients whose PM_10_ exposure level fell in the second tertile had a 30% higher likelihood of having pneumonia than that of first tertile patients, and the risk for those who were in the third tertile was almost doubled.

**Conclusion:**

The consistent findings toward a positive association between PM_10_ levels and the likelihood of experiencing pneumonia due to Covid-19 make the implementation of new strategies to reduce air pollution more and more urgent.

**Supplementary Information:**

The online version contains supplementary material available at 10.1186/s12889-021-10949-9.

## Background

Covid-19 infection, whose pathogenic agent is Severe Acute Respiratory Syndrome Coronavirus 2 (SARS-CoV-2), was firstly reported in Wuhan, Hubei Province, China, in December 2019 [1]. The spectrum of Covid-19 clinical manifestation is very wide, as it includes asymptomatic infection, mild upper respiratory tract symptoms, mild and severe pneumonia, acute respiratory distress syndrome, sepsis and septic shock, [2] but also myalgia, fatigue, sputum production, headache, hemoptysis, diarrhea, anosmia, and ageusia. Furthermore, as clinical data became available, potential cardiovascular, gastrointestinal, neurological, and cutaneous manifestations of the disease were described by scientific literature [3]. Despite the course of the disease is often mild and undistinguishable from a common flu, in a considerable number of cases Covid-19 infection may require hospitalization, and can lead to an acute respiratory distress syndrome (ARDS) and death [1] [4]. Covid-19 outbreak was officially confirmed as a pandemic on 11 February 2020, [5] and since the end of February 2020, contagious has rapidly spread in Italy, particularly in the North (Lombardy, Veneto and Emilia-Romagna), and in many other European countries. As of the end of March, Italy represented the third country worldwide in terms of total number of cases and the first one in terms of total number of deaths [6]. As of March 2021, it is estimated that more than 3 million people have contracted Covid-19, and more than 100,000 persons died as a consequence of this infection in Italy. Regions where most of the new cases and deaths are reported are still those of Northern Italy, and particularly Lombardy [7]. A large part of Northern Italy territory includes the Po basin area (Padan Plain), which is the most industrialized area of the country, and one of the most polluted region of Europe [8]. The World Health Organization (WHO), reported that air pollution is responsible for 7 million deaths worldwide every year, and represented one of the main concern regarding public health [9]. Airborne particulate matter (PM), which is a heterogeneous mixture of solid and liquid, organic and inorganic material suspended in air, is considered as the most relevant component of air pollution [10].

A growing body of evidences is showing that some countries were subject to a greater spread of Covid-19 and suffered higher lethality, and this undoubtably captured researchers attention [11]. As a consequence, several studies have been focusing on the potential role of atmospheric pollutants, particularly PM, in the diffusion of Covid-19 both in the short- and the long-term, as well as in the impact of the virus on human health [5, 11–46]. One of the ideas underneath the potential relationship between airborne PM and Covid-19 diffusion is that the atmospheric PM might exercise a carrier action along with the virus [5, 26]. Setti and colleagues recently demonstrated the presence of the SARS-CoV-2 RNA on PM [47]. Furthermore, it has been hypothesised that the presence of air-related pollutants can put pressure on the health conditions of the populations at risk, thus offering preconditions for the development of Covid-19 and its complications, including life-threatening ones [11]. Almost all of the studies so far performed succeeded in finding a positive association between air pollution and Covid-19 diffusion and its health-related outcomes. However, it is worth mentioning that most of them were ecological studies, thus relied on aggregated data only. Differently, the analysis here reported used patient-level data. In particular, data available from the Italian Regional Environmental Protection Agencies (ARPA) on ≤10 μm diameter PM (PM_10_) daily concentrations were collected to assign an individual exposure to a sample of patients experiencing Covid-19 infection. Patients’ data on Covid-19 was retrieved from IQVIA® Longitudinal Patient Database (LPD), a large repository of secondary data fed by approximately 900 Italian General Practitioners (GPs).

To authors’ knowledge, this was the first study ever using patient-level data aimed at estimating the association between short-term exposure to PM_10_ and the likelihood of experiencing pneumonia due to Covid-19 infection as a proxy of disease severity on a large sample of patients in Italy.

## Methods

### Data sources

Information on Covid-19 patients was retrieved from the Italian IQVIA® LPD database. Italian IQVIA® LPD is part of a computerized network of GPs from different European countries feeding a centralized database with extensive and anonymous data on patients’ consultations and treatments. This database reflects the clinical practice of a national sample of GPs since it allows the collection and longitudinal analysis of data taken from patients’ records related to prescriptions and healthcare resource utilisations in everyday clinical practice. Drug prescriptions and medical diagnoses are both coded directly by GPs and comply with the Anatomical Therapeutic and Chemical (ATC) classification system, and with the 9th edition of International Classification of Disease (ICD-9-CM), respectively. Currently, about 900 Italian GPs contribute to the IQVIA® LPD, providing data from routinely collected records of ~ 1.2 million patients. The Italian IQVIA® LPD database, established in 1998 by the Italian College of General Practitioners (Società Italiana di Medicina Generale - SIMG), was found to be representative of the Italian general population [48–50] and a reliable source of information in numerous previous studies for several disease areas [51–57].

PM_10_ daily concentration data detected by the official air quality monitoring stations located on the entire Italian territory were retrieved from ARPA Regional websites for the period January 2020 – June 2020 and are publicly available.

### Study population, exposure, and outcomes definitions

On March 17^th^, 2020, all the GPs collaborating with the Italian IQVIA® LPD were asked by SIMG/HealthSearch to use two different codes to distinguish between Covid-19 patients with or without pneumonia. The present analysis firstly selected all patients who had at least one Covid-19 registration during the period March 18^th^, 2020 – June 30^th^, 2020 (selection period). The date of the first Covid-19 registration was considered as the Index Date and patients were grouped based on the presence/absence of a registration of Covid-19 with pneumonia within 90 days since the Index Date (follow-up) as a proxy of disease severity. The final study cohort was composed of Covid-19 patients who had data availability for the study period and no missing information on age, sex, and PM_10_ exposure. For each patient included in the analysis, diagnoses recorded during the 12-month period preceding the Index Date (baseline) were collected, and the following conditions, defined through ICD-9 codes, were considered as comorbidities of interest: hypertension (ICD-9 code 401.xx), diabetes (ICD-9 code 250.xx), asthma (ICD-9 code 493.xx), chronic obstructive pulmonary disease (COPD) (ICD-9 codes 490.xx, 491.xx, 492.xx, 494.xx, 495.xx, 496.xx), obesity (ICD-9 codes 278.00, 278.01 and/or a body mass index value of at least 30 kg/m^2^), coronary artery disease (CAD) (ICD-9 codes 410.xx, 411.xx, 412.xx, 413.xx, 414.xx, V45.81, V45.82), and cerebrovascular disease (ICD-9 codes from 430.xx to 438.xx). Information on age and sex on the Index Date, smoking habits, and GP’s office province were also retrieved from the IQVIA® LPD database. The daily average value of PM_10_ (μg/m^3^) was calculated at province level based on data from ARPA air quality monitoring stations. Individual PM_10_ exposure was then assigned to each patient by calculating the mean of PM_10_ daily values observed during the month preceding the Index Date and according to GP’s office province.

### Statistical analysis

A description of patients’ individual PM_10_ exposure was given in terms of mean and standard deviation by Region. Descriptive statistics on patients’ characteristics were reported for the two groups of patients with or without pneumonia. A stratification of patients by tertiles of PM_10_ calculated on the overall cohort and presence or absence of pneumonia was also provided. Because the two groups of Covid-19 patients with or without pneumonia were independent, when dealing with categorical variables, Chi-square tests were performed to investigate on between-groups differences. Because of the large sample size, Kolmogorov-Smirnov test was performed to test normality of PM_10_ exposure assessed as a continuous variable. *P*-value resulting from the test was very close to 0 (i.e., < 0.01), thus a non-parametric Wilcoxon signed-rank test was performed to understand whether PM_10_ exposure differed between the two groups. *P*-values resulting from Chi-square and Wilcoxon tests were reported as continuous variables. As *p*-values can range from 0 to 1, the closer the values were to zero, the higher the diffidence in the null hypothesis of no association [58]. Given the multilevel structure of the data, a multiple mixed-effects logistic regression model was built by inserting a random intercept for GPs’ office province to assess the association between PM_10_ exposure and the likelihood of experiencing pneumonia as a consequence of Covid-19 infection. Indeed, no further details in addition to province were available regarding GPs’ offices location, and then on patients’ residence. Being so, PM_10_ values were used only at province level, with this possibly introducing within-province correlation despite an individual level of exposure was assigned to each patient depending on his/her Index Date. Furthermore, due to the pandemic, the disease status of subjects in the same province may be correlated, violating the independence assumption of the ordinary regression model [59]. Fixed effects included in the model were PM_10_ exposure in terms of tertiles, sex, age class, and presence or absence of each comorbidity of interest during baseline period. Given the large number of patients involved, we used the maximum likelihood as an estimation method. Covariance structure was assumed to be variance component and the ratio between the Pearson chi-square statistic and its degrees of freedom was calculated to verify that variability has been properly modeled. Associations were expressed as Odds Ratios (ORs), together with their 95% confidence intervals (CI). Point estimates should be regarded as the most compatible values, while the other values included in the CIs should be regarded as progressively less compatible (but nevertheless still compatible) the greater their distance from the point estimate [58]. Multiple logistic mixed models have been previously used in literature to deal with data having an analogous structure to investigate on the association between air pollution and health outcomes [59–61]. Sensitivity analyses were performed to assess the robustness of results. Firstly, we run the above described model on the subgroup of subjects with smoking habits information available to account for the potential confounding effect of cigarettes smoking on the association between exposure to PM_10_ and risk of pneumonia. A dummy variable indicating whether a patient have ever smoked or not was included among model’s covariates. Secondly, we decided to run the multiple mixed-effects logistic regression model using PM_10_ tertiles calculated based on patients’ individual exposure during the 14- and 7-day periods preceding the Index Date as exposure variables. The latter analyses were intended to test the robustness of results with respect to the time-window chosen to assess PM_10_ exposure. Thirdly, stratified analyses were performed by running the multiple mixed-effects logistic regression model separately on men and women, on < 65 and ≥ 65 years old subjects, and on never smoking patients. C-statistic was calculated to assess models’ discrimination performance (i.e., the extent to which patients who were predicted to be high risk exhibited higher pneumonia rates compared with those who were predicted to be low risk); C-statistic can range from 0.5 (poor discrimination) to 1.0 (perfect discrimination) and was used in previous studies as a measure of model’s discrimination ability [62]. All the analyses were performed using SAS software, version 9.4.

## Results

**Fig. 1:**
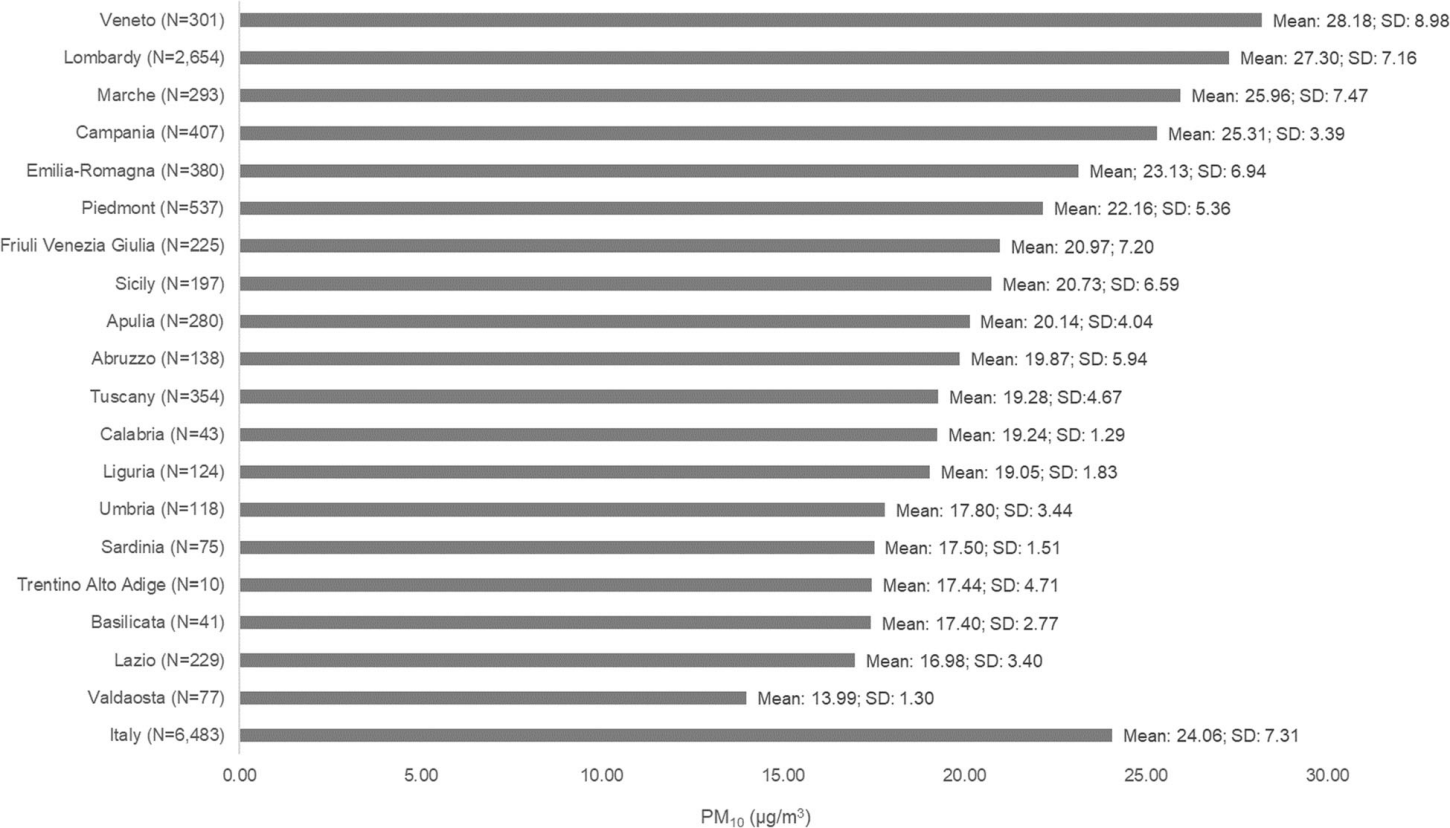
PM_10_ (μg/m^3^) - Mean (standard deviation) values calculated based on the average daily values during the month preceding the Index Date for each patient by Region

A final cohort of 6483 patients was defined according to eligibility criteria. Overall, Covid-19 patients without pneumonia numbered 5404 (83.4%), while those with pneumonia were 1079 (16.6%). Figure 1, which describes individual PM_10_ exposure by Region, shows that on average, Veneto, Lombardy, Marche, and Campania were the Regions where patients have been exposed to the highest level of PM_10_ during the month preceding the Index Date. In particular, PM_10_ mean values ranged from 25.3 to 28.2 μg/m^3^, thus being higher than the mean value calculated on the total cohort (24.1 μg/m^3^). For all the remaining Regions, PM_10_ mean values fell below the Italian mean value and ranged from 14.0 μg/m^3^ for Valdaosta to 23.1 μg/m^3^ for Emilia-Romagna (Fig. 1). Molise Region was not included in the analysis due to IQVIA® LPD data unavailability.

Table 1 reports Covid-19 patients’ characteristics stratified by pneumonia presence or absence. Among Covid-19 patients with pneumonia we found a higher proportion of male than the one observed among patients without pneumonia (57.1% versus 45.9%). Overall, mean age of Covid-19 patients was around 55 years, and subjects with pneumonia were older than those without pneumonia, with mean age being around 64 and 53 years respectively for the two groups (data not shown). Consistently, the proportion of Covid-19 patients aged 65 years or older was 50.4 and 26.9% for the group with and without pneumonia, respectively. Ex- and current smokers accounted for a higher proportion among Covid-19 patients without pneumonia. Overall, the most frequently reported comorbidities among those of interest were hypertension, obesity, and diabetes mellitus. The comparison between groups showed that among pneumonia patients the proportions of subjects with each condition were higher than those observed among patients without pneumonia. PM_10_ exposure during the month preceding the Index Date measured as a continuous variable was higher for pneumonia patients (Table 1).

**Table 1:** Characteristics of study patients by presence/absence of pneumonia

	Total (***N*** = 6483)		COVID-19 without pneumonia (***N*** = 5404)		COVID-19 with pneumonia (***N*** = 1079)		
***Patients characteristics***							**p-value**
**Gender**							<.0001
Male: N (%)	3095	(47.74%)	2479	(45.87%)	616	(57.09%)	
**Age classes**							<.0001
14 ≤ Age < 45 years: N (%)	1781	(27.47%)	1672	(30.94%)	109	(10.10%)	
45 ≤ Age < 55 years: N (%)	1387	(21.39%)	1200	(22.21%)	187	(17.33%)	
55 ≤ Age < 65 years: N (%)	1316	(20.30%)	1076	(19.91%)	240	(22.24%)	
65 ≤ Age < 75 years: N (%)	963	(14.85%)	743	(13.75%)	220	(20.39%)	
Age ≥ 75 years	1036	(15.98%)	713	(13.19%)	323	(29.97%)	
**Smoking status**^**a**^							<.0001
Never smoked: N (%)	3635	(86.24%)	2987	(85.22)	648	(91.27%)	
Ever smoked: N (%)	580	(13.76%)	518	(14.78%)	62	(8.73%)	
**Comorbidities**							
Hypertension: N (%)	1821	(28.09%)	1405	(26.00%)	416	(38.55%)	<.0001
Diabetes: N (%)	537	(8.28%)	388	(7.18%)	149	(13.81%)	<.0001
Asthma: N (%)	319	(4.92%)	252	(4.66%)	67	(6.21%)	0.0320
Obesity^b^: N (%)	765	(11.80%)	594	(10.99%)	171	(15.85%)	<.0001
COPD: N (%)	420	(6.48%)	323	(5.98%)	97	(8.99%)	0.0002
CAD: N (%)	297	(4.58%)	220	(4.07%)	77	(7.14%)	<.0001
CVD: N (%)	309	(4.77%)	236	(4.37%)	73	(6.77%)	0.0007
**Comorbidities number**							
0: N (%)	3664	(56.52%)	3200	(59.22%)	465	(43.00%)	<.0001
1: N (%)	1652	(25.48%)	1340	(24.80%)	312	(28.92%)	
2: N (%)	791	(12.20%)	585	(10.83%)	206	(19.09%)	
3+: N (%)	376	(5.80%)	279	(5.16%)	97	(8.99%)	
**PM**_**10**_^c^							
Mean (SD)	24.06	(7.31)	23.59	(7.22)	26.40	(7.32)	<.0001
Median (Q1; Q3)	23.40	(17.78; 30.38)	22.94	(17.52; 29.74)	28.56	(20.30; 32.45)	

*SD* standard deviation; *Q1* first quartile; *Q3* third quartile

^a^ Calculated on 4215 subjects with smoking habits available information

^b^ Body Mass Index > 30 kg/m^2^ or ICD-9 in 278.0x, 278.00, 278.01

^c^ PM_10_ exposure during the month preceding the Index Date measured as a continuous variable

**Fig. 2:**
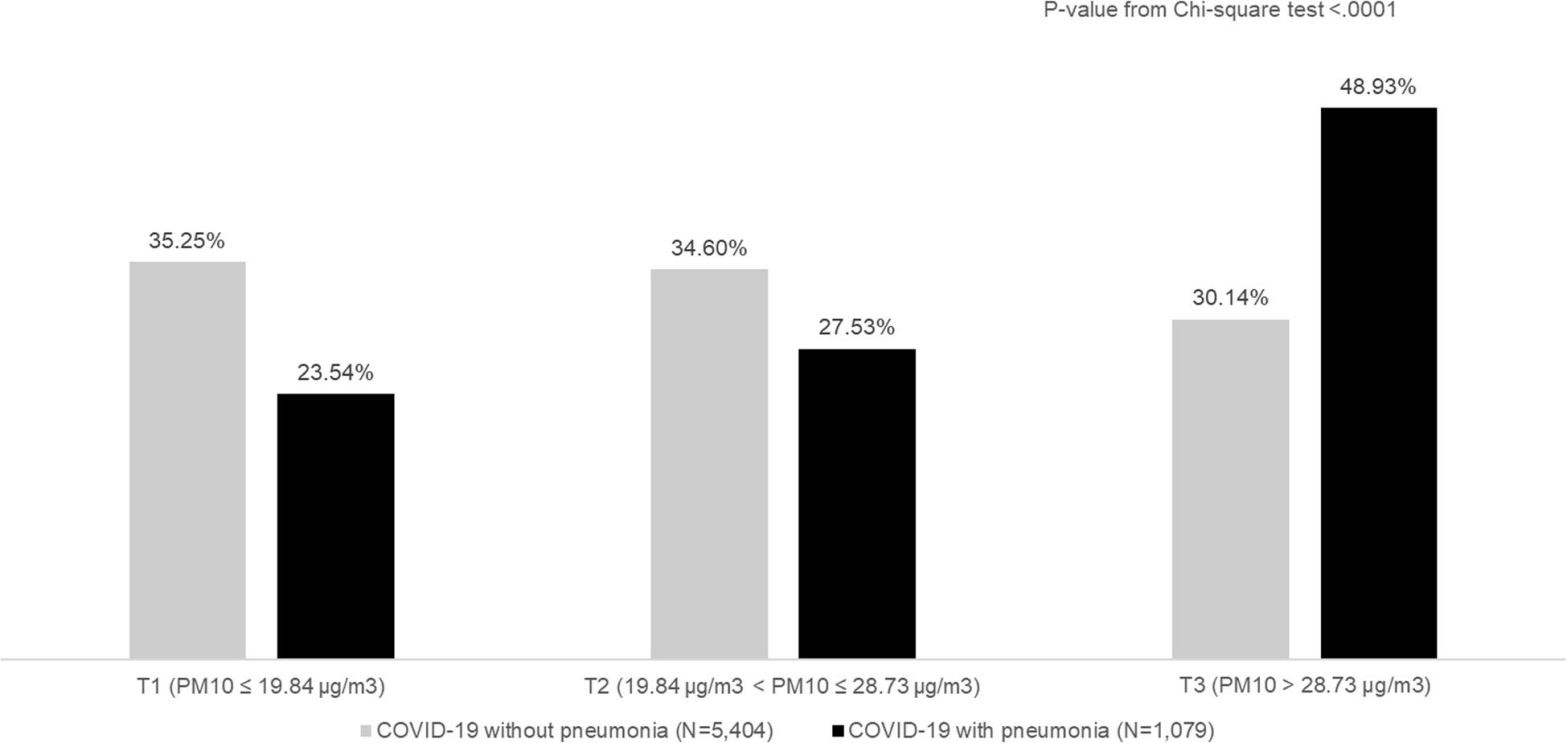
COVID-19 patients stratified by presence/absence of pneumonia and tertiles of PM_10_ exposure during the month preceding the Index Date

The stratification of Covid-19 patients by presence/absence of pneumonia and tertiles of PM_10_ exposure showed that a much higher proportion of subjects whose mean level of exposure fell in the third tertile was found for pneumonia group. Indeed, almost half of patients with pneumonia had a PM_10_ mean value higher than 28.7 μg/m^3^ during the month preceding the Index Date, i.e., Covid-19 diagnosis (Fig. 2).

Results from the multiple mixed-effect logistic regression model showed that PM_10_ exposure level during the month preceding the Index Date was associated with the risk of experiencing pneumonia due to Covid-19 infection. In particular, patients whose level of PM_10_ exposure fell in the second tertile had a 30% higher likelihood of having pneumonia than patients whose level of exposure fell into the first tertile; the risk for patients in the third PM_10_ exposure tertile was almost doubled. Among the covariates included in the model, gender, age class, and presence/absence of baseline asthma and or obesity had an influence on the likelihood of experiencing pneumonia. In particular, male sex, older age, as well as the presence of asthma and/or obesity exposed patients to a higher risk of pneumonia as a consequence of Covid-19 infection. The ratio of the Pearson chi-square statistic and its degrees of freedom was 0.96. The C-statistic was 0.8, thus discrimination performance of the model was good (Table 2).

**Table 2:** Estimates from the multiple mixed-effect logistic regression model evaluating the likelihood of experiencing pneumonia

Characteristic	Category	Odds Ratio	95% Confidence Interval	
			**Lowe Limit**	**Upper Limit**
**PM**_**10**_ **tertiles**	PM_10_ ≤ 19.84 μg/m^3^	1.00	–	–
	19.84 μg/m^3^ < PM_10_ ≤ 28.73 μg/m^3^	1.34	1.09	1.65
	PM_10_ > 28.73 μg/m^3^	1.93	1.55	2.39
**Gender**	Female	1.00	–	–
	Male	1.52	1.31	1.76
**Age class**	14 ≤ Age < 45 years: N (%)	1.00	–	–
	45 ≤ Age < 55 years: N (%)	2.25	1.74	2.91
	55 ≤ Age < 65 years: N (%)	3.09	2.40	3.99
	Age ≥ 65 years	5.51	4.29	7.06
**Hypertension**	No	1.00	–	–
	Yes	0.99	0.84	1.17
**Diabetes**	No	1.00	–	–
	Yes	1.23	0.97	1.55
**Asthma**	No	1.00	–	–
	Yes	1.58	1.16	2.16
**COPD**	No	1.00	–	–
	Yes	1.02	0.78	1.34
**Coronary artery disease**	No	1.00	–	–
	Yes	0.96	0.71	1.31
**Cerebrovascular disease**	No	1.00	–	–
	Yes	1.01	0.75	1.37
**Obesity**	No	1.00	–	–
	Yes	1.24	1.01	1.54

C = 0.77

Results from the sensitivity analysis run on the subgroup of patients with available information on smoking habits confirmed those from the model run on the total cohort and did not show any association between smoking habits and risk of pneumonia (data not shown). Furthermore, results from the multiple mixed-effect logistic regression models which accounted for PM_10_ exposure assessed during the 14- and 7-day periods preceding the Index Date (Tables 1S and 2S), as well as those from stratified analyses (Tables 3S, 4S, 5S, 6S, 7S) confirmed the robustness of our findings.

## Discussion

The main objective of the present study was to understand whether short-term exposure to PM_10_ may increase Covid-19 patients’ likelihood of experiencing pneumonia as a proxy of disease severity. Findings from the multiple mixed-effect logistic model suggest that short-term exposure to PM_10_ may represent a risk factor for the development of pneumonia in patients with Covid-19 infection. Furthermore, an increasing trend in the likelihood of experiencing pneumonia was observed corresponding to increasing levels of PM_10_.

Several studies previously investigated on the association between air pollution and Covid-19 spread and adverse outcomes in Italy [4, 8, 10, 11, 13–15, 29, 30, 35, 36, 40, 44, 46]. A study by Conticini and colleagues focused on two Northern Italy Regions, Lombardy and Emilia Romagna, which are part of the Padan plain. According to data from Italian Civil Protection, these Regions had the highest level of virus lethality in the world at the time of the first epidemic wave [63]. Being so, Conticini and colleagues speculated that the high level of pollution should be considered as an additional co-factor of the high level of lethality recorded in that area [4]. Dettori and colleagues examined the role of air pollutants in relation to the number of deaths per each Italian province affected by Covid-19. PM_10_ was found to be an independent predictor for Covid-19-related mortality [11]. Similarly, Bianconi et al. investigated on the association between PM exposure and Covid-19 cases and related deaths both at Regional and province level in Italy. Study results seemed to suggest that the greater diffusion and lethality of Covid-19 might be at least partially related to the past and cumulative PM exposure [10]. Accarino and colleagues, who investigated on short-term exposure to atmospheric pollutants and spatio-temporal distribution of Covid-19 cases and deaths in Italy, also suggested a potential correlation, particularly with PMs [35]. Coker and colleagues found that a 1 μg/m^3^ increase in long-term exposure to PM_2.5_ was associated with a 9% increase in COVID-19 related mortality in Northern Italy [29]. Similar studies have been conducted also in other countries [17, 20, 23, 25, 27, 30, 33, 34, 36, 38, 39, 41–43, 45]. A study by Wu and colleagues investigating on the association between PM_2.5_ exposure and risk of COVID-19 death in the United States found that an increase of 1 μg/m^3^ in PM_2.5_ was associated with an 8% increase in the COVID-19 death rate [20]. Cole and colleagues found a positive relationship between air pollution and Covid-19 cases, hospital admissions and deaths using data from 355 Dutch municipalities [23].

Findings from all the above-mentioned analyses came from ecological studies, which used aggregated data. Undoubtably, ecological studies, whose approach is extremely cost-effective, are crucial in rapidly evolving areas of research. Indeed, they allow drawing area level conclusions, which can be useful for policy-making [64]. However, ecological regression analyses are unable to adjust for individual-level risk factors, which, instead, are known to affect Covid-19 health outcomes. To authors’ knowledge, very few studies have been performed using individual-level data, and none of them was conducted in Italy. Travaglio and colleagues investigated on the associations between several air pollutants and the risk of COVID-19 infection using patient-level data obtained from a cohort of 1450 subjects in the UK. Results from the analysis showed that levels of PM pollutants and nitrogen oxides were associated with an increase in SARS-CoV-2 infections. No investigations were done accounting for Covid-19 severity or mortality [32]. Another study conducted in the UK and using individual-level data found a positive association between exposure to NO_2_ and Covid-19 mortality, while the association with PM_2.5_ was uncertain [31]. Finally, a study conducted in Mexico City used patient-level data to estimate the effects of both long- and short-term exposure to PM_2.5_ on Covid-19 mortality: evidences toward a positive relationship between PM_2.5_ air pollution and the likelihood for an individual to die following Covid-19 infection did emerge; this relationship increased with age, and, although findings suggested that the association was mainly driven by long-term exposure, authors did not exclude that short-term exposure might also have an effect [37].

In light of the few studies using patient-level data, authors of the present study do believe that findings here reported should be regarded as extremely significant and add an important contribution in the understanding of the relationship between air pollution and Covid-19 severity. Furthermore, it is worth mentioning that individual-level data in the present study included information on exposure in addition to that on potential confounders. In fact, PM_10_ exposure was calculated for each patient considering a specific time-window defined based on Covid-19 registration date. Moreover, the inclusion of data from the entire Italian territory and the extension of the study period up to the end of June, allowed to account for a very high variability in terms of exposure. Besides this, important strengths of the present study are the representativeness of the data source used to identify Covid-19 cases [49], as well as the setting where data was collected. Indeed, GPs are on the front-line in the management of this pandemic as they are the first point of contact for people affected by Covid-19, except for those patients who develop extremely severe forms of the disease since its onset. As such, we were able to retrieve information on a representative sample of patients in terms of disease severity, and this prevented our study from the risk of selection bias which may have occurred using inpatient-setting data. Risk factors for Covid-19-related pneumonia other than PM_10_ exposure identified by the present study were older age, male sex, asthma, and obesity. These findings agree with those from previous studies, with this further confirming the robustness of our data. In particular, Polverino et al. found that 65 years older age and male sex were among predictors of death in a sample of Covid-19 inpatients [65]. Similarly, increasing age was one of the independent risk factors for all-cause in-hospital mortality in a study conducted on 317 hospitalized adult patients with a diagnosis of Covid-19 [66]. Baronio and colleagues found that admission to intensive care unit (ICU) and poor survival were associated with advanced age and higher body mass index [67]. Furthermore, obesity was found to be a strong, independent risk factor for respiratory failure, admission to the ICU and death in a sample of 482 Covid-19 hospitalized patients [68]. Finally, a study conducted on behalf of the National Health System (NHS) in England linked primary-care electronic medical records of 17,278,392 adults to 10,926 Covid-19-related deaths; among factors associated with Covid-19 death there were male sex, greater age, and severe asthma [69].

The present study also presents some limits. Firstly, we do not know whether Covid-19 diagnoses were confirmed by a nasopharyngeal swab. However, demographic characteristics of the overall cohort are in line with Covid-19 cases description provided by the Italian Istituto Superiore di Sanità (ISS). Indeed, according to ISS data, the proportion of men among subjects affected by Covid-19 as of the end of June 2020 was 45.8%, compared to the 47.7% observed in the present study. Also, age class distributions were in line, even if patients in the present study were just slightly younger [70]. However, it should be considered that the selection period for the present study was delayed with respect to Covid-19 outbreak, and, differently from sex distribution which remained constant, mean age of Covid-19 patients has progressively decreased [70]. In light of the above consideration, we do believe that IQVIA® LPD is a reliable data source for the identification of Covid-19 cases. Secondly, the main analysis investigating on the association between PM_10_ exposure and risk of Covid-19-related pneumonia performed on the overall cohort did not account for smoking habits due to the limited availability of such information. However, results from the sensitivity analysis run on the subgroup of patients confirmed all the associations found by the analysis performed on the overall cohort. Furthermore, it is worth mentioning that there were previous studies that did not find a correlation between smoking and adverse outcomes in Covid-19 patients [71]. Thirdly, it is possible that the date of the Covid-19 diagnosis registration did not exactly correspond to the date of infection’s onset. However, authors of the present study are confident that the application of a 30-day time-window to estimate average PM_10_ exposure should have mitigated the potential effect of this limitation. Finally, PM_10_ comprises PM_2.5_ and PM_5_, and may also contain a heterogeneous group of gaseous compounds such as sulfur dioxide (SO_2_) and nitrogen dioxide (NO_2_) [72]. As such, it cannot be excluded that the adverse effect of PM_10_ on Covid-19 outcomes we observed might be attributed to one or more of PM_10_ components, instead of PM_10_ itself. This is the reason why authors of the present study would like to claim the importance of reducing air pollution as a whole.

## Conclusion

This is the first study aimed to investigate on the association between PM_10_ exposure and the risk of developing Covid-19-related pneumonia as a proxy of disease severity using individual-level data in Italy. Consistent findings toward a positive association between PM_10_ levels and the likelihood of experiencing pneumonia were found. Authors of the present study would like to claim the urgency of implementing new strategies to reduce air pollution.

## Supplementary Information

**Additional file 1.** Table 1S – Estimates from the multiple mixed-effect logistic regression model evaluating the likelihood of experiencing pneumonia. Sensitivity analysis with PM_10_ exposure assessed during the 14-day period preceding the Index Date (i.e., date of first Covid-19 registration). Table 2S – Estimates from the multiple mixed-effect logistic regression model evaluating the likelihood of experiencing pneumonia. Sensitivity analysis with PM_10_ exposure assessed during the 7-day period preceding the Index Date (i.e., date of first Covid-19 registration). Table 3S – Estimates from the multiple mixed-effect logistic regression model evaluating the likelihood of experiencing pneumonia in men. Table 4S – Estimates from the multiple mixed-effect logistic regression model evaluating the likelihood of experiencing pneumonia in women. Table 5S – Estimates from the multiple mixed-effect logistic regression model evaluating the likelihood of experiencing pneumonia in 65 years younger subjects. Table 6S – Estimates from the multiple mixed-effect logistic regression model evaluating the likelihood of experiencing pneumonia in subjects aged ≥65 years. Table 7S – Estimates from the multiple mixed-effect logistic regression model evaluating the likelihood of experiencing pneumonia in never smoking subjects.

## Data Availability

IQVIA® LPD data that support the findings of this study are available from IQVIA, but restrictions apply to the availability of these data, which were used under license for the current study, and so are not publicly available. Data are, however, available from the corresponding author upon reasonable request and with permission of IQVIA. PM_10_ daily concentration data detected by the official air quality monitoring stations located on the entire Italian territory were retrieved from ARPA Regional websites for the period January 2020 – June 2020 and are publicly available.
