# Supplementary material for: An Italian individual-level data study investigating on the association between air pollution exposure and Covid-19 severity in primary-care setting
Source: BMC Public Health. 2021 May 12;21:902. doi: 10.1186/s12889-021-10949-9 (PMC8114667; doi:10.1186/s12889-021-10949-9)
Supplement: Supplementary file 1 — Additional file 1. Table 1S – Estimates from the multiple mixed-effect logistic regression model evaluating the likelihood of experiencing pneumonia. Sensitivity analysis with PM10 exposure assessed during the 14-day period preceding the Index Date (i.e., date of first Covid-19 registration). Table 2S – Estimates from the multiple mixed-effect logistic regression model evaluating the likelihood of experiencing pneumonia. Sensitivity analysis with PM10 exposure assessed during the 7-day period preceding the Index Date (i.e., date of first Covid-19 registration). Table 3S – Estimates from the multiple mixed-effect logistic regression model evaluating the likelihood of experiencing pneumonia in men. Table 4S – Estimates from the multiple mixed-effect logistic regression model evaluating the likelihood of experiencing pneumonia in women. Table 5S – Estimates from the multiple mixed-effect logistic regression model evaluating the likelihood of experiencing pneumonia in 65 years younger subjects. Table 6S – Estimates from the multiple mixed-effect logistic regression model evaluating the likelihood of experiencing pneumonia in subjects aged ≥65 years. Table 7S – Estimates from the multiple mixed-effect logistic regression model evaluating the likelihood of experiencing pneumonia in never smoking subjects. [file 12889_2021_10949_MOESM1_ESM.docx]

Table 1S – Estimates from the multiple mixed-effect logistic regression model evaluating the likelihood of experiencing pneumonia. Sensitivity analysis with PM_10_ exposure assessed during the 14-day period preceding the Index Date (i.e., date of first Covid-19 registration)

| **Characteristic** | | **Category** | **Odds Ratio** | **95% Confidence Interval** | |
| --- | --- | --- | --- | --- | --- |
|  | |  |  | **Lowe Limit** | **Upper Limit** |
| **PM_10_ tertiles** | PM_10_ ≤ 17.83 µg/m^3^ | | 1.00 | - | - |
|  | 17.83 µg/m^3^ < PM_10_ ≤ 27.25 µg/m^3^ | | 1.36 | 1.12 | 1.65 |
|  | PM_10_ > 27.25 µg/m^3^ | | 1.65 | 1.35 | 2.00 |
|  |  | |  |  |  |
| **Gender** | Female | | 1.00 | - | - |
|  | Male | | 1.52 | 1.31 | 1.76 |
|  |  | |  |  |  |
| **Age class** | 14 ≤ Age < 45 years: N (%) | | 1.00 | - | - |
|  | 45 ≤ Age < 55 years: N (%) | | 2.26 | 1.74 | 2.92 |
|  | 55 ≤ Age < 65 years: N (%) | | 3.09 | 2.40 | 3.98 |
|  | Age ≥ 65 years | | 5.44 | 4.24 | 6.97 |
|  |  | |  |  |  |
| **Hypertension** | No | | 1.00 | - | - |
|  | Yes | | 0.99 | 0.84 | 1.17 |
|  |  | |  |  |  |
| **Diabetes** | No | | 1.00 | - | - |
|  | Yes | | 1.23 | 0.98 | 1.56 |
|  |  | |  |  |  |
| **Asthma** | No | | 1.00 | - | - |
|  | Yes | | 1.58 | 1.16 | 2.15 |
|  |  | |  |  |  |
| **COPD** | No | | 1.00 | - | - |
|  | Yes | | 1.03 | 0.79 | 1.35 |
|  |  | |  |  |  |
| **Coronary artery disease** | No | | 1.00 | - | - |
|  | Yes | | 0.97 | 0.71 | 1.32 |
|  |  | |  |  |  |
| **Cerebrovascular disease** | No | | 1.00 | - | - |
|  | Yes | | 1.00 | 0.74 | 1.36 |
|  |  | |  |  |  |
| **Obesity** | No | | 1.00 | - | - |
|  | Yes | | 1.26 | 1.02 | 1.55 |

C=0.77

Table 2S – Estimates from the multiple mixed-effect logistic regression model evaluating the likelihood of experiencing pneumonia. Sensitivity analysis with PM_10_ exposure assessed during the 7-day period preceding the Index Date (i.e., date of first Covid-19 registration)

| **Characteristic** | | **Category** | **Odds Ratio** | **95% Confidence Interval** | |
| --- | --- | --- | --- | --- | --- |
|  | |  |  | **Lowe Limit** | **Upper Limit** |
| **PM_10_ tertiles** | PM_10_ ≤ 17.61 µg/m^3^ | | 1.00 | - | - |
|  | 17.61 µg/m^3^ < PM_10_ ≤ 26.29 µg/m^3^ | | 1.25 | 1.04 | 1.52 |
|  | PM_10_ > 26.29 µg/m^3^ | | 1.51 | 1.24 | 1.83 |
|  |  | |  |  |  |
| **Gender** | Female | | 1.00 | - | - |
|  | Male | | 1.52 | 1.31 | 1.76 |
|  |  | |  |  |  |
| **Age class** | 14 ≤ Age < 45 years: N (%) | | 1.00 | - | - |
|  | 45 ≤ Age < 55 years: N (%) | | 2.28 | 1.76 | 2.95 |
|  | 55 ≤ Age < 65 years: N (%) | | 3.10 | 2.40 | 3.99 |
|  | Age ≥ 65 years | | 5.43 | 4.23 | 6.96 |
|  |  | |  |  |  |
| **Hypertension** | No | | 1.00 | - | - |
|  | Yes | | 1.00 | 0.84 | 1.17 |
|  |  | |  |  |  |
| **Diabetes** | No | | 1.00 | - | - |
|  | Yes | | 1.23 | 0.97 | 1.55 |
|  |  | |  |  |  |
| **Asthma** | No | | 1.00 | - | - |
|  | Yes | | 1.58 | 1.16 | 2.15 |
|  |  | |  |  |  |
| **COPD** | No | | 1.00 | - | - |
|  | Yes | | 1.04 | 0.79 | 1.36 |
|  |  | |  |  |  |
| **Coronary artery disease** | No | | 1.00 | - | - |
|  | Yes | | 0.97 | 0.72 | 1.32 |
|  |  | |  |  |  |
| **Cerebrovascular disease** | No | | 1.00 | - | - |
|  | Yes | | 1.01 | 0.74 | 1.36 |
|  |  | |  |  |  |
| **Obesity** | No | | 1.00 | - | - |
|  | Yes | | 1.26 | 1.02 | 1.56 |

C=0.77

Table 3S – Estimates from the multiple mixed-effect logistic regression model evaluating the likelihood of experiencing pneumonia in men.

| **Characteristic** | | **Category** | **Odds Ratio** | **95% Confidence Interval** | |
| --- | --- | --- | --- | --- | --- |
|  | |  |  | **Lowe Limit** | **Upper Limit** |
| **PM_10_ tertiles** | PM_10_ ≤ 19.71 µg/m^3^ | | 1.00 | - | - |
|  | 19.71 µg/m^3^ < PM_10_ ≤ 28.91 µg/m^3^ | | 1.32 | 1.00 | 1.74 |
|  | PM_10_ > 28.91 µg/m^3^ | | 2.14 | 1.60 | 2.86 |
|  |  | |  |  |  |
| **Age class** | 14 ≤ Age < 45 years: N (%) | | 1.00 | - | - |
|  | 45 ≤ Age < 55 years: N (%) | | 3.10 | 2.13 | 4.51 |
|  | 55 ≤ Age < 65 years: N (%) | | 4.55 | 3.17 | 6.54 |
|  | Age ≥ 65 years | | 7.19 | 5.01 | 10.34 |
|  |  | |  |  |  |
| **Hypertension** | No | | 1.00 | - | - |
|  | Yes | | 0.97 | 0.78 | 1.21 |
|  |  | |  |  |  |
| **Diabetes** | No | | 1.00 | - | - |
|  | Yes | | 1.32 | 0.98 | 1.79 |
|  |  | |  |  |  |
| **Asthma** | No | | 1.00 | - | - |
|  | Yes | | 2.03 | 1.29 | 3.20 |
|  |  | |  |  |  |
| **COPD** | No | | 1.00 | - | - |
|  | Yes | | 0.91 | 0.64 | 1.30 |
|  |  | |  |  |  |
| **Coronary artery disease** | No | | 1.00 | - | - |
|  | Yes | | 0.90 | 0.63 | 1.30 |
|  |  | |  |  |  |
| **Cerebrovascular disease** | No | | 1.00 | - | - |
|  | Yes | | 0.94 | 0.63 | 1.40 |
|  |  | |  |  |  |
| **Obesity** | No | | 1.00 | - | - |
|  | Yes | | 1.17 | 0.88 | 1.55 |

C=0.77

Table 4S – Estimates from the multiple mixed-effect logistic regression model evaluating the likelihood of experiencing pneumonia in women.

| **Characteristic** | | **Category** | **Odds Ratio** | **95% Confidence Interval** | |
| --- | --- | --- | --- | --- | --- |
|  | |  |  | **Lowe Limit** | **Upper Limit** |
| **PM_10_ tertiles** | PM_10_ ≤ 19.86 µg/m^3^ | | 1.00 | - | - |
|  | 19.86 µg/m^3^ < PM_10_ ≤ 28.59 µg/m^3^ | | 1.32 | 0.97 | 1.80 |
|  | PM_10_ > 28.59 µg/m^3^ | | 1.74 | 1.27 | 2.39 |
|  |  | |  |  |  |
| **Age class** | 14 ≤ Age < 45 years: N (%) | | 1.00 | - | - |
|  | 45 ≤ Age < 55 years: N (%) | | 1.67 | 1.17 | 2.39 |
|  | 55 ≤ Age < 65 years: N (%) | | 2.03 | 1.40 | 2.93 |
|  | Age ≥ 65 years | | 4.54 | 3.20 | 6.44 |
|  |  | |  |  |  |
| **Hypertension** | No | | 1.00 | - | - |
|  | Yes | | 0.98 | 0.76 | 1.27 |
|  |  | |  |  |  |
| **Diabetes** | No | | 1.00 | - | - |
|  | Yes | | 1.10 | 0.75 | 1.62 |
|  |  | |  |  |  |
| **Asthma** | No | | 1.00 | - | - |
|  | Yes | | 1.27 | 0.82 | 1.99 |
|  |  | |  |  |  |
| **COPD** | No | | 1.00 | - | - |
|  | Yes | | 1.31 | 0.86 | 2.01 |
|  |  | |  |  |  |
| **Coronary artery disease** | No | | 1.00 | - | - |
|  | Yes | | 1.17 | 0.65 | 2.12 |
|  |  | |  |  |  |
| **Cerebrovascular disease** | No | | 1.00 | - | - |
|  | Yes | | 1.13 | 0.71 | 1.82 |
|  |  | |  |  |  |
| **Obesity** | No | | 1.00 | - | - |
|  | Yes | | 1.33 | 0.96 | 1.85 |

C=0.78

Table 5S – Estimates from the multiple mixed-effect logistic regression model evaluating the likelihood of experiencing pneumonia in 65 years younger subjects.

| **Characteristic** | | **Category** | **Odds Ratio** | **95% Confidence Interval** | |
| --- | --- | --- | --- | --- | --- |
|  | |  |  | **Lowe Limit** | **Upper Limit** |
| **PM_10_ tertiles** | PM_10_ ≤ 19.87 µg/m^3^ | | 1.00 | - | - |
|  | 19.87 µg/m^3^ < PM_10_ ≤ 28.87 µg/m^3^ | | 1.27 | 0.96 | 1.67 |
|  | PM_10_ > 28.87 µg/m^3^ | | 1.89 | 1.43 | 2.50 |
|  |  | |  |  |  |
| **Gender** | Female | | 1.00 | - | - |
|  | Male | | 1.62 | 1.34 | 1.97 |
|  |  | |  |  |  |
| **Hypertension** | No | | 1.00 | - | - |
|  | Yes | | 1.20 | 0.93 | 1.54 |
|  |  | |  |  |  |
| **Diabetes** | No | | 1.00 | - | - |
|  | Yes | | 1.93 | 1.27 | 2.93 |
|  |  | |  |  |  |
| **Asthma** | No | | 1.00 | - | - |
|  | Yes | | 1.58 | 1.07 | 2.31 |
|  |  | |  |  |  |
| **COPD** | No | | 1.00 | - | - |
|  | Yes | | 1.11 | 0.67 | 1.84 |
|  |  | |  |  |  |
| **Coronary artery disease** | No | | 1.00 | - | - |
|  | Yes | | 1.44 | 0.73 | 2.84 |
|  |  | |  |  |  |
| **Cerebrovascular disease** | No | | 1.00 | - | - |
|  | Yes | | 1.72 | 0.90 | 3.30 |
|  |  | |  |  |  |
| **Obesity** | No | | 1.00 | - | - |
|  | Yes | | 1.47 | 1.10 | 1.97 |

C=0.73

Table 6S – Estimates from the multiple mixed-effect logistic regression model evaluating the likelihood of experiencing pneumonia in subjects aged ≥ 65 years.

| **Characteristic** | | **Category** | **Odds Ratio** | **95% Confidence Interval** | |
| --- | --- | --- | --- | --- | --- |
|  | |  |  | **Lowe Limit** | **Upper Limit** |
| **PM_10_ tertiles** | PM_10_ ≤ 19.45 µg/m^3^ | | 1.00 | - | - |
|  | 19.45 µg/m^3^ < PM_10_ ≤ 28.22 µg/m^3^ | | 1.34 | 0.98 | 1.83 |
|  | PM_10_ > 28.22 µg/m^3^ | | 2.29 | 1.67 | 3.15 |
|  |  | |  |  |  |
| **Gender** | Female | | 1.00 | - | - |
|  | Male | | 1.43 | 1.14 | 1.79 |
|  |  | |  |  |  |
| **Hypertension** | No | | 1.00 | - | - |
|  | Yes | | 1.03 | 0.82 | 1.29 |
|  |  | |  |  |  |
| **Diabetes** | No | | 1.00 | - | - |
|  | Yes | | 1.11 | 0.83 | 1.48 |
|  |  | |  |  |  |
| **Asthma** | No | | 1.00 | - | - |
|  | Yes | | 1.45 | 0.85 | 2.49 |
|  |  | |  |  |  |
| **COPD** | No | | 1.00 | - | - |
|  | Yes | | 1.07 | 0.77 | 1.49 |
|  |  | |  |  |  |
| **Coronary artery disease** | No | | 1.00 | - | - |
|  | Yes | | 0.98 | 0.69 | 1.39 |
|  |  | |  |  |  |
| **Cerebrovascular disease** | No | | 1.00 | - | - |
|  | Yes | | 0.91 | 0.64 | 1.29 |
|  |  | |  |  |  |
| **Obesity** | No | | 1.00 | - | - |
|  | Yes | | 1.04 | 0.76 | 1.42 |

C=0.76

Table 7S – Estimates from the multiple mixed-effect logistic regression model evaluating the likelihood of experiencing pneumonia in never smoking subjects.

| **Characteristic** | | **Category** | **Odds Ratio** | **95% Confidence Interval** | |
| --- | --- | --- | --- | --- | --- |
|  | |  |  | **Lowe Limit** | **Upper Limit** |
| **PM_10_ tertiles** | PM_10_ ≤ 20.10 µg/m^3^ | | 1.00 | - | - |
|  | 20.10 µg/m^3^ < PM_10_ ≤ 29.39 µg/m^3^ | | 1.39 | 1.06 | 1.81 |
|  | PM_10_ > 29.39 µg/m^3^ | | 2.05 | 1.56 | 2.70 |
|  |  | |  |  |  |
| **Gender** | Female | | 1.00 | - | - |
|  | Male | | 1.55 | 1.28 | 1.88 |
|  |  | |  |  |  |
| **Age class** | 14 ≤ Age < 45 years: N (%) | | 1.00 | - | - |
|  | 45 ≤ Age < 55 years: N (%) | | 2.38 | 1.61 | 3.51 |
|  | 55 ≤ Age < 65 years: N (%) | | 3.30 | 2.26 | 4.82 |
|  | Age ≥ 65 years | | 5.87 | 4.05 | 8.51 |
|  |  | |  |  |  |
|  |  | |  |  |  |
| **Hypertension** | No | | 1.00 | - | - |
|  | Yes | | 1.01 | 0.82 | 1.25 |
|  |  | |  |  |  |
| **Diabetes** | No | | 1.00 | - | - |
|  | Yes | | 1.31 | 1.00 | 1.72 |
|  |  | |  |  |  |
| **Asthma** | No | | 1.00 | - | - |
|  | Yes | | 2.27 | 1.54 | 3.35 |
|  |  | |  |  |  |
| **COPD** | No | | 1.00 | - | - |
|  | Yes | | 1.08 | 0.77 | 1.52 |
|  |  | |  |  |  |
| **Coronary artery disease** | No | | 1.00 | - | - |
|  | Yes | | 0.96 | 0.66 | 1.40 |
|  |  | |  |  |  |
| **Cerebrovascular disease** | No | | 1.00 | - | - |
|  | Yes | | 1.02 | 0.71 | 1.47 |
|  |  | |  |  |  |
| **Obesity** | No | | 1.00 | - | - |
|  | Yes | | 1.32 | 1.04 | 1.68 |

C=0.78
